# Weight trends in a multiethnic cohort of pediatric acute lymphoblastic leukemia survivors: A longitudinal analysis

**DOI:** 10.1371/journal.pone.0217932

**Published:** 2019-05-31

**Authors:** Kayla L. Foster, Kathleen D. Kern, Tiffany M. Chambers, Philip J. Lupo, Kala Y. Kamdar, Michael E. Scheurer, Austin L. Brown

**Affiliations:** 1 St. Jude Children’s Research Hospital, Memphis, Tennessee, United States of America; 2 Baylor College of Medicine, Houston, Texas, United States of America; 3 Texas Children’s Cancer and Hematology Centers, Houston, Texas, United States of America; University of Bern, SWITZERLAND

## Abstract

**Background:**

As survival rates for childhood acute lymphoblastic leukemia (ALL) continue to improve, there is growing concern over the chronic health conditions that survivors face. Given that survivors of childhood ALL are at increased risk of cardiovascular complications and obesity, we sought to characterize BMI trends from diagnosis through early survivorship in a multi-ethnic, contemporary cohort of childhood ALL patients and determine if early weight change was predictive of long-term weight status.

**Methods:**

The study population consisted of ALL patients aged 2–15 years at diagnosis who were treated with chemotherapy alone at Texas Children’s Hospital. Each patient had BMI z-scores collected at diagnosis, 30-days post-diagnosis, and annually for five years. Linear regression models were estimated to evaluate the association between: 1) BMI z-score change in the first 30 days and BMI z-scores at five-years post-diagnosis; and 2) BMI z-score change in the first year post-diagnosis and BMI z-scores at five-years post-diagnosis.

**Results:**

This retrospective cohort study included longitudinal data from 121 eligible patients. The mean BMI z-scores for the population increased significantly (p-value<0.001) from baseline (mean = 0.25) to 30 days post-diagnosis (mean = 1.17) before plateauing after one year post-diagnosis (mean = 0.99). Baseline BMI z-scores were statistically significant predictors to five year BMI z-scores (p <0.001). Independent of baseline BMI z-score and other clinical factors, the BMI z-score at one year post-diagnosis was significantly associated with BMI z-score at five-years post-diagnosis (β = 0.63, p <0.001), while BMI z-score at 30 days post-diagnosis was not (β = 0.10, p = 0.23).

**Conclusion:**

Our results suggest that weight gain within the first year after diagnosis is more strongly associated with long-term BMI than early weight gain (within 30 days). If confirmed, this information may help identify a window of time during therapy when ALL patients would benefit most from weight management directed interventions.

## Introduction

Acute lymphoblastic leukemia (ALL) is the most common childhood malignancy, with more than 5,000 cases diagnosed annually in the United States [1]. With the emergence of more effective chemotherapeutic protocols, ALL is curable in more than 90% of cases [2]. As survival has increased, research focus has shifted to long-term health outcomes of ALL survivors. Unfortunately, curative therapy is associated with a burden of chronic health conditions among survivors, with 95.5% of child and adolescent cancer survivors experiencing at least one chronic disease by 45 years of age [3, 4]. Compared to age-matched peers, ALL survivors have a four-fold increased risk of mortality secondary to cardiovascular events [5]. Because obesity plays a major role in cardiovascular health, approaches that minimize treatment-related weight gain may improve the overall health of childhood ALL patients and survivors [6–8].

The U.S. Preventive Services Task Force (USPSTF) recommends screening body mass index (BMI) in all children ≥6 years of age and offering metabolic screening to high risk individuals [9]. While height and weight are routinely monitored at pediatric oncology visits, there is no defined increase in BMI that warrants intervention within the ALL population. A 2015 meta-analysis by Zhang et al. described longitudinal changes in BMI for ALL patients [8]. This study identified increases in weight during the early treatment phase, which persisted into survivorship. However, few studies have systematically evaluated weight trajectories during treatment in populations large enough to identify at which point weight gain becomes predictive of long-term weight status. Moreover, ethnic minorities such as Hispanics are underrepresented in the current literature, despite Hispanics being disproportionally affected by both childhood ALL and childhood obesity compared to non-Hispanic white children [10, 11]. Therefore, the aim of this study is to characterize BMI trends in a multi-ethnic cohort of childhood ALL patients during therapy and determine whether early changes in BMI on treatment were associated with post-treatment weight status.

## Methods

In this retrospective cohort study, subjects were those diagnosed with ALL at Texas Children’s Hospital between 2005 and 2012. To reduce heterogeneity in treatment exposures, patients were excluded if they relapsed, received bone marrow transplant, or were exposed to cranial or craniospinal irradiation therapy (CRT). In addition, patients with conditions that might impact growth trajectories (e.g., Down syndrome, dwarfism) were also excluded. Finally, the study analysis was restricted to patients two to 15 years of age at diagnosis, thereby permitting the calculation of BMI z-scores for each individual at each study visit. This study was approved by the Baylor College of Medicine Institutional Review Board (IRB).

All variables/factors assessed for this study were abstracted from medical records. Patients included in this analysis were treated on or according to the following Children’s Oncology Group (COG) or Pediatric Oncology Group (POG) ALL protocols: AALL0232, AALL0331, AALL0932, AALL1131, POG 9904, POG 9905. Detailed treatment information for each protocol can be found at www.clinicaltrials.gov/ [12]. Briefly, pediatric ALL treatment generally lasts two to three years and consists of three treatment phases: 1) remission induction with a combination of vincristine, daunomycin, asparaginase, methotrexate and a corticosteroid chemotherapy; 2) intensification with a combination of asparaginase, vincristine, mercaptopurine, methotrexate, doxorubicin, cytarabine, and corticosteroids; and 3) maintenance therapy with a combination of methotrexate, vincristine, and corticosteroids.

Height and weight data collected during clinic visits were recorded for seven time points: diagnosis, 30-day post diagnosis, and annually 1- to 5-years post diagnosis. BMI was calculated from recorded height and weight at each visit. BMI z-scores were estimated from age- and gender-specific 2000 Centers for Disease Control and Prevention (CDC) growth charts for individuals aged 2–20 years and research participants were categorized into three CDC-recommended groups: underweight/normal (BMI z-score < 1.0365), overweight (BMI z-score: 1.0365–1.645), and obese (BMI z-score ≥ 1.645) [13]. Additional covariates were also abstracted from medical records, including race/ethnicity (Non-Hispanic white; Non-Hispanic black; Hispanic), age at diagnosis, and gender.

Descriptive statistics were calculated for the overall study population and compared across underweight/normal weight, overweight, and obese BMI z-score categories determined at five years post-diagnosis. We applied latent class growth analysis (LCGA) to characterize average BMI z-score trajectory groups from baseline to 5 years. To find the best model fit, three, four, and five latent class models were tested using both linear and quadratic polynomial terms. After comparing the overall p-value and Bayesian information criterion (BIC) value between each model, the model with three latent classes was identified as the best statistical fit. Separate multivariable linear regressions models were used to assess the relationship between the final BMI z-score and (1) change in BMI z-scores between diagnosis and 30-days post-diagnosis and (2) change in BMI z-scores between diagnosis and one year post-diangosis, adjusting for patient demographic and clinical characteristics. Covariates were selected for inclusion in multivariable regression models using a backward stepwise approach, with the final model including statistically significant or clinically meaningful variables (e.g., age, sex, race). Associations between the final BMI z-score and independent variables were deemed significant if the p-value < 0.05. All data was analyzed using Stata 14.0 (StataCorp LP, College Station, TX).

## Results

**Fig 1:**
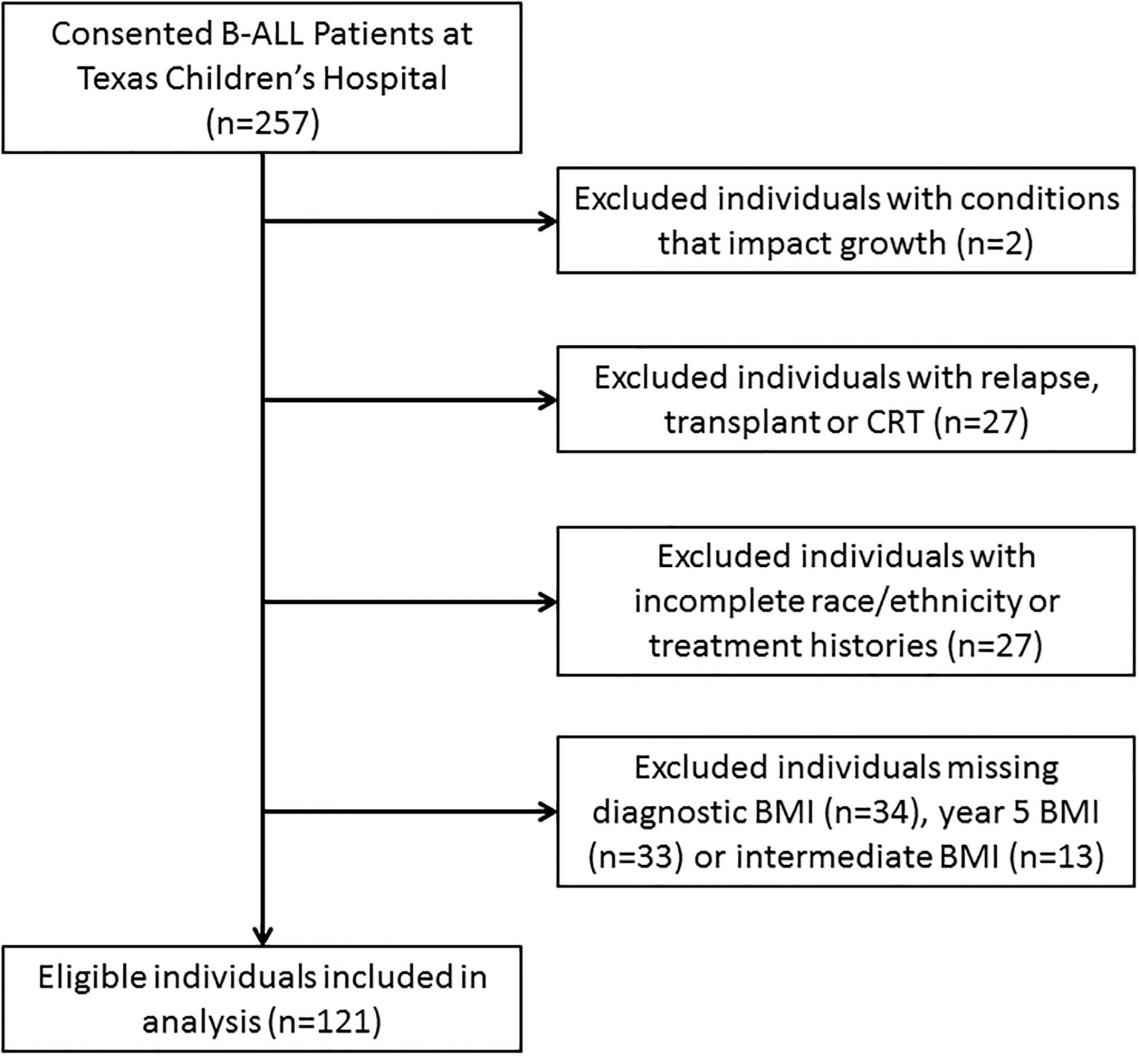
Flow diagram of patient eligibility.

We identified a total of 121 eligible patients diagnosed with B-cell ALL and treated at Texas Children’s Hospital between 2005 and 2012 (Fig 1). The study population was 51% male (n = 62), 56% diagnosed before the age of five (n = 68), and comprised of 58% Hispanic (n = 70) and 36% non-Hispanic white (n = 44) patients (Table 1). Most patients were treated on or according to AALL0331 (64%). The distribution of demographic factors was similar between patients excluded from the analysis due to missing or incomplete data and patients included in the analysis (results not shown): age (p-value = 0.33), race/ethnicity (p-value = 0.97), and gender (p-value = 0.75).

**Table 1:** Demographic and clinical characteristics of pediatric ALL patients.

		zBMI Class at 5-years Post-diagnosis		
	Overall (n = 121)	Underweight/Normal (n = 53)	Overweight (n = 26)	Obese (n = 42)
Age at Diagnosis, mean (SD)	5.52 (3.13)	5.65 (3.32)	5.29 (2.71)	5.48 (3.18)
zBMI at Diagnosis, mean (SD)	0.25 (1.35)	-0.51 (1.10)	0.23 (1.17)	1.23 (1.09)
zBMI at 5-years, mean (SD)	0.99 (1.13)	-0.05 (0.81)	1.29 (0.19)	2.12 (0.32)
zBMI Class at Diagnosis, n(%)				
Underweight/Normal	85 (70.2)	49 (92.5)	21 (80.8)	15 (35.7)
Overweight	18 (14.9)	4 (7.6)	2 (7.7)	12 (28.6)
Obese	18 (15.9)	0 (0.0)	3 (11.5)	15 (35.7)
Race/Ethnicity, n(%)				
Non-Hispanic White	44 (36.4)	21 (39.6)	11 (42.3)	12 (28.6)
Non-Hispanic Black	7 (5.8)	4 (7.6)	1 (3.9)	2 (4.8)
Hispanic	70 (57.9)	28 (52.8)	14 (53.9)	28 (66.7)
Gender, n(%)				
Male	62 (51.2)	27 (50.9)	14 (53.8)	21 (50.0)
Female	59 (48.8)	26 (49.1)	12 (46.2)	21 (50.0)
Induction Glucocorticoid, n(%)				
Dexamethasone	103 (85.1)	47 (88.7)	23 (88.5)	33 (78.6)
Prednisone	18 (14.9)	6 (11.3)	3 (11.5)	9 (21.4)
Mean Cumulative Dose, (SD)				
Asparaginase (IU/m2)	7777 (5601)	8354 (6574)	7200 (4525)	7436 (4950)
Cyclophosphamide (g/m2)	1.7 (1.0)	1.7 (1.1)	1.5 (0.9)	1.8 (1.0)
Cytarabine IV (g/m2)	1.3 (2.0)	1.6 (3.0)	1.1 (0.7)	1.1 (0.6)
Cytarabine IT (mg/m2)	76.9 (65.9)	67.3 (15.1)	66.5 (7.8)	94.3 (106.5)
Dexamethasone (g/m2)	1.1 (0.4)	1.1 (0.4)	1.0 (0.3)	1.1 (0.4)
Doxorubicin (mg/m2)	78.9 (17.3)	82.8 (23.2)	75.0 (0.0)	76.3 (12.8)
Mercaptopurine (g/m2)	57.7 (18.6)	57.2 (17.1)	54.5 (24.2)	60.4 (16.5)
Methotrexate PO (g/m2)	1.8 (0.7)	1.8 (0.7)	1.5 (0.7)	1.9 (0.6)
Methotrexate IV (g/m2)	3.7 (5.5)	2.7 (2.4)	3.4 (5.6)	5.3 (7.1)
Methotrexate IT (mg/m2)	243.6 (65.1)	255.3 (78.7)	231.2 (45.0)	237.2 (55.9)
Prednisone (g/m2)	3.8 (1.3)	3.4 (1.0)	3.6 (0.8)	4.1 (1.7)
Thioguanine (g/m2)	0.9 (0.7)	1.0 (1.1)	0.9 (0.2)	0.8 (0.0)
Vincristine (mg/m2)	56.6 (13.5)	56.6 (12.5)	55.3 (10.1)	57.4 (16.4)

ALL, acute lymphoblastic leukemia; zBMI, Body Mass Index z-score; SD, standard deviation; PO, per os; IV, intravenous; IT, intrathecal

**Fig 2:**
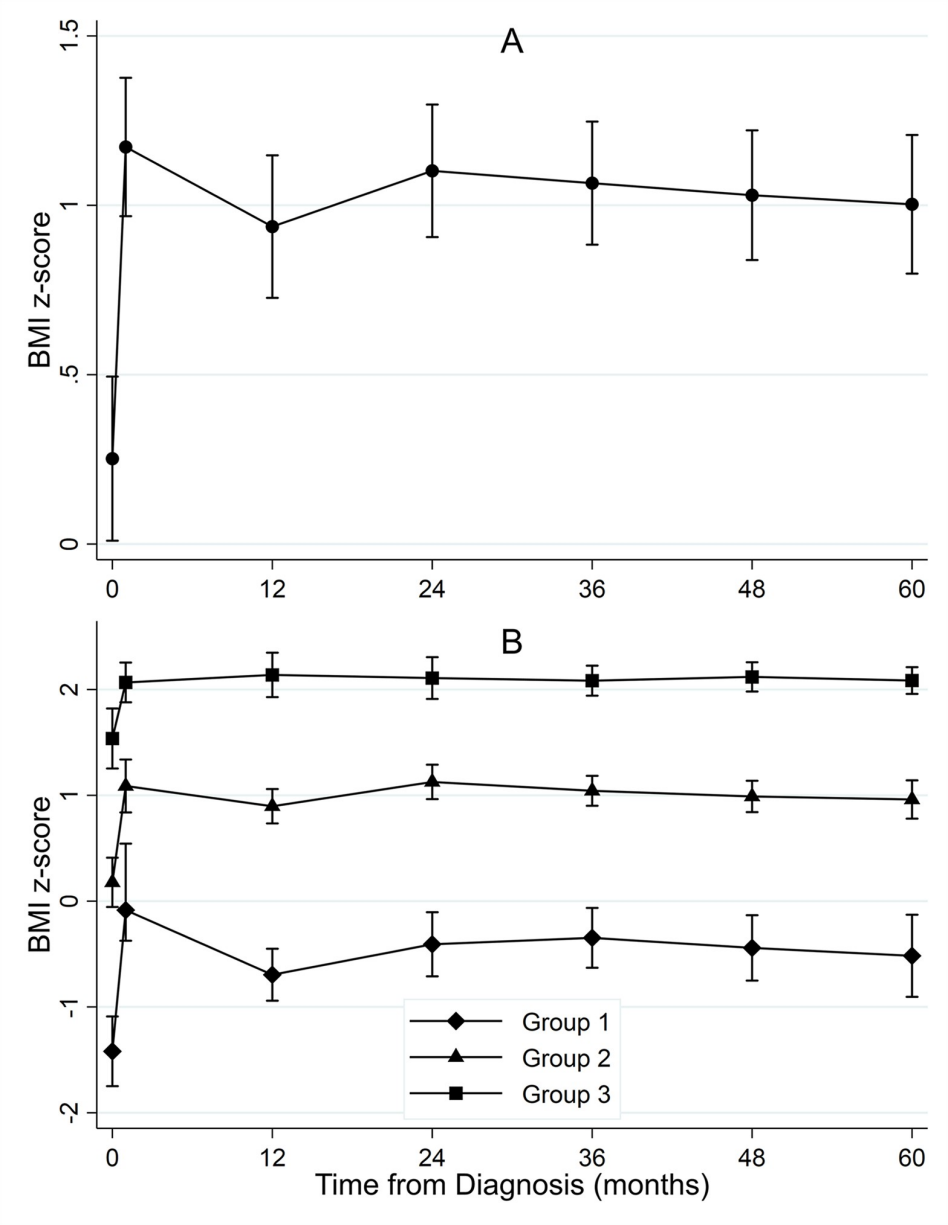
Mean trajectory of BMI z-scores (A) and latent class growth analysis of BMI z-scores (B) between diagnosis and five years post-diagnosis in 121 pediatric ALL patients.

The majority (n = 85, 70%) of patients were normal weight (n = 72, 60%) or underweight (n = 13, 11%) at diagnosis. Conversely, most patients were either overweight (n = 26, 22%) or obese (n = 42, 35%) by five years post-diagnosis. Between diagnosis and five years post-diagnosis (Fig 2A), the mean BMI z-score increased from 0.25 (range: -2.76, 3.40) to 0.99 (range: -2.33, 2.83), corresponding to a statistically significant (p-value = 2.5x10^-14^) mean increase of 0.74 standard deviations (range: -1.65, 5.44).

LCGA identified three latent classes of BMI z-score trajectories between diagnosis and five years post-diagnosis (Fig 2B), with 21% of patients (n = 25, group 1) classified in a low BMI z-score trajectory (mean BMI z-score at diagnosis = -0.66), 50% (n = 60, group 2) classified in a moderate BMI z-score trajectory (mean BMI z-score at diagnosis = 0.75), and 30% (n = 36, group 3) classified in a high BMI z-score trajectory (mean BMI z-score at diagnosis = 1.87). On average, the mean BMI z-scores for each group increased between diagnosis and five years post-diagnosis, with a best-fit linear trend for change reaching statistical significance in group 2 (p-value = 0.0047) and group 3 (p-value = 0.0499). Mean BMI z-scores increased rapidly in the first 30 days of treatment for each group (overall mean increase = 0.92 SD, 95% CI: 0.68–1.16): group 1 mean increase = 1.50 SD (95% CI: 1.00–2.01), group 2 mean increase = 0.91 SD (95% CI: 0.53–1.29), group 3 mean increase = 0.53 SD (95% CI: 0.21–0.85). Overall increases in BMI z-scores were attenuated slightly by one year post-diagnosis but remained significantly elevated from diagnosis (overall mean increase from diagnosis = 0.69 SD, 95% CI: 0.51–0.86), particularly in the low BMI z-score trajectory (group 1, mean increase from diagnosis = 0.72, 95% CI: 0.34–1.11). BMI z-scores remained relatively stable beyond one year post-diagnosis, increasing only slightly (overall mean increase between one and five years post-diagnosis = 0.05, 95% CI: -0.10–0.19). The mean BMI z-score change between one and five years post-diagnosis did not reach statistical significance in any of the three groups.

In the model evaluating 30 day change in BMI z-scores (Table 2), only BMI z-score at diagnosis (β = 0.58, 95% CI: 0.43–0.74) was a statistically significant (p-value = 5.2x10^-11^) predictor of BMI z-scores at five years post-diagnosis, with higher BMI z-scores at diagnosis associated with higher BMI z-scores five years later. In the second regression model, both BMI z-scores at diagnosis (β = 0.76, 95% CI: 0.65–0.88) and change in BMI z-scores between diagnosis and one year were statistically significant predictors of BMI z-scores at five years. Each SD increase in BMI z-scores between diagnosis and one year was associated with a 0.63 SD increase (95% CI: 0.47–0.79) in five year BMI z-scores, independent of age, sex, race/ethnicity, and BMI z-scores at diagnosis. No other variables evaluated in multivariable regression models, including age, sex, race/ethnicity, glucocorticoid type, or treatment protocol, were significantly associated with BMI z-scores at five years post-diagnosis.

**Table 2:** Association between clinical and demographic factors and BMI z-score at 5 years post-diagnosis among pediatric ALL patients.

	Model 1: 30 day zBMI change		Model 2: 1 year zBMI change	
	β (95% CI)	P-value	β (95% CI)	P-value
zBMI change, z-score	0.10 (-0.07–0.27)	0.233	0.63 (0.47–0.79)	<0.001
zBMI at Diagnosis, z-score	0.58 (0.43–0.74)	<0.001	0.76 (0.65–0.88)	<0.001
Age at Diagnosis, year	-0.01 (-0.07–0.04)	0.666	0.00 (-0.04–0.05)	0.876
Ethnicity				
Non-Hispanic White	Ref		Ref	
Non-Hispanic Black	0.10 (-0.63–0.83)	0.779	-0.24 (-0.84–0.36)	0.433
Hispanic	0.15 (-0.19–0.49)	0.384	0.11 (-0.17–0.38)	0.455
Gender				
Male	Ref		Ref	
Female	-0.01 (-0.25–0.37)	0.961	0.01 (-0.26–0.28)	0.953

ALL, acute lymphoblastic leukemia; zBMI, body mass index z-score; CI, confidence interval. Models were adjusted for other covariates listed in the table

## Discussion

This longitudinal study of a contemporary multi-ethnic cohort of childhood ALL patients demonstrates that increases in weight are common during therapy and persist into survivorship. This finding adds to the growing body of evidence that weight gain is a common consequence of modern pediatric ALL chemotherapy, even among patients spared CRT [14–17]. Few, if any, assessments of obesity in childhood ALL patients have evaluated weight change during treatment windows that predict post-treatment adiposity. Specifically, although change in BMI z-scores was most pronounced during the first 30 days of therapy, BMI z-score at 30 days was not a statistically significant predictor of BMI z-scores at five years post-diagnosis after accounting for clinical and demographic factors. Weight often peaked after 30 days, but did not return to pre-treatment levels. Importantly, weight maintained at one year post-diagnosis did correlate with weight maintained at five years post-diagnosis. This observation has potential clinical implications for monitoring and intervening on weight gain during childhood ALL therapy.

Obesity is a well-recognized consequence of childhood ALL therapy among survivors treated with CRT [18, 19]; however, recent publications have highlighted the obesogenic nature of contemporary childhood ALL chemotherapy. In particular, prolonged exposure to corticosteroids has been linked to increased energy intake and weight gain during childhood ALL treatment [14, 20, 21], although the association between corticosteroids and long-term adiposity among survivors is not well-established [5, 22]. Other chemotherapeutic agents may contribute to adverse BMI profiles by adversely impacting energy expenditure by interfering with cardiovascular or motor-sensory function [23, 24]. Our results are consistent with recent research demonstrating that, on average, ALL patients gain weight rapidly during the first 30 days of therapy, which correlates with high dose steroids given during remission induction therapy [14, 25–27]. Other studies have also observed decreases in BMI z-scores between the end of induction (approximately 30 days post-diagnosis) and maintenance therapy [14, 25–27], although this observation was not apparent among patients with elevated BMI z-scores at diagnosis in our study, suggesting patients with lower diagnosis BMI z-scores might be more resistant to initial weight gain during induction therapy. Notably, beyond one year post-diagnosis, BMI z-scores remained relatively stable, consistent with previous reports [27–29]. Additional research is needed to fully evaluate the impact of weight gain during treatment on long-term cardiovascular and metabolic outcomes in survivors of childhood ALL. However, given that childhood ALL therapy is associated with an increased risk of chronic conditions later in life which may be further exacerbated by excess weight, metabolic and BMI screening are likely important components of a comprehensive survivorship care plan for childhood ALL survivors. This is particularly true for patients with elevated BMI z-scores at diagnosis, as these patients were most likely to be overweight or obese throughout treatment and early survivorship.

This study provides new insight into trends in BMI z-scores in a multi-ethnic population of patients being treated for childhood ALL. At diagnosis, the prevalence of overweight and obesity in the study population (30%) was similar to recent estimates of the prevalence among 2–19 year olds in the general population [30]. Consistent with disparities in overweight and adiposity among unaffected children, we observed difference in the proportion of overweight and obese children between Hispanic (37%) and non-Hispanic white (20%) patients at diagnosis. By 5-years post-diagnosis the prevalence of overweight and obesity increased to 56% of the study population, including 60% of Hispanics and 52% of non-Hispanic whites. These estimates are markedly higher the population-based prevalence of 32% overall, 39% among Hispanics, and 29% among non-Hispanics whites [30]. A number of social, cultural, and environmental factors contribute to ethnic disparities in childhood obesity in the general population and might be deserving of additional attention in future studies of patients with childhood ALL [11].

Our findings must be considered in the light of certain limitations. First, the ALL survivors included in this study were only monitored consistently for a minimum of five years from diagnosis. Despite this limitation, one advantage to this study is that the patients were treated with contemporary protocols, which reduces influence of CRT or heterogeneity of chemotherapeutic regimens over time. Our population also reflects one of the most diverse studied to date, with >60% being Hispanic or non-Hispanic black, thereby improving our ability to generalize these findings to previously understudied populations. Notably, the present study may have been underpowered with the available sample size to detect meaningful difference in weight trajectories by race or ethnicity. Moreover, we do not yet have the data to support that weight and BMI at five years post-diagnosis are predictive of long-term adult BMI, although existing data suggests post-diagnosis BMI remained fairly steady for at least seven years [27]. Presumably, our population would follow similar growth patterns, but additional longitudinal studies are needed to determine patterns in obesity and related comorbidities amongst contemporary ALL survivors. Finally, our analysis was restricted to individuals with complete information and we only evaluated potential explanatory variables that were ascertainable from patient medical records. While we did not observe evidence that patients excluded from the analysis differed meaningfully from patients included in the analysis, we cannot rule out potential selection bias. Additional studies are needed to evaluate other potentially important predictors of obesity in childhood ALL patients, including eating disorders, endocrine or growth disruptions, psychosocial outcomes, and socioeconomic factors.

This study highlights that, even in the absence of CRT, treatment-related weight gain remains a concern for childhood ALL patients. Given that excess adiposity is a poor prognostic factors for leukemia relapse and survival [31], effective weight management in this population may improve both treatment efficacy and long-term cardiovascular health [31, 32]. Weight loss interventions specific to ALL survivors and pediatric oncology patients have not yet been established. In a systematic review by the USPSTF [9], medium to high intensity comprehensive behavioral interventional programs, consisting of more than 26 hours of contact time, were shown to be the most effective approach to weight loss in pediatric obesity. Regular interaction with a primary pediatrician or other health professional that routinely monitors growth in this population is likely necessary to achieve this, especially amongst a population where treating active malignancy is the priority. A recent study of pediatric ALL recommended that interventions to prevent obesity begin during induction therapy, particularly for those with obesity or short stature at the time of diagnosis. Using a multidisciplinary approach and engaging the treating oncologist, primary care physician, nurses, dietitians, physical therapists, and psychologists from the start of therapy would likely lead to the best outcomes with regard to prevention of treatment related obesity [14].

Current general pediatrics guidelines advocate for the consideration of laboratory evaluation of children with a BMI percentile > 85% and risk factors for obesity-related health conditions [33, 34]. Given the high prevalence of obesity observed among survivors of childhood ALL [35, 36], rapid weight gain during obesogenic childhood ALL therapy may have similar health implications and should be considered an indication to screen and intervene. Even if programs are established to prevent obesity during ALL treatment, regular and frequent screenings will remain essential. The results of this study suggest that the one year time point be an appropriate time to screen for significant BMI changes. If BMI z-scores are stably elevated one year post-diagnosis, further cardiovascular screening and secondary obesity prevention may be warranted.
